# Early Mortality Rate and Associated Risk Factor in Patients Undergoing Primary Total Hip Replacement at a Tertiary Hospital in Tanzania

**DOI:** 10.1155/aort/4831975

**Published:** 2025-03-26

**Authors:** Disha Deograthias Wadosa, Violet Lupondo, Adam Hussein, Jimmy Olomi

**Affiliations:** ^1^Department of Orthopedics and Traumatology, Massana Hospital and College of Nursing, Dar es Salaam, Tanzania; ^2^Department of Orthopedics and Traumatology, Muhimbili University of Health and Allied Sciences, Dar-es-Salam, Tanzania; ^3^Department of Orthopedics and Traumatology, Muhimbili Orthopedics Institute, Dar-es-Salam, Tanzania; ^4^Department of Orthopedics and Traumatology, University of Dar es Salaam, Mbeya, Tanzania

## Abstract

**Background:** Hip joint replacement surgery or total hip arthroplasty (THA) is an effective procedure for elderly patients. It can improve their quality of life and functionality while reducing the direct costs associated with arthritis. With increased THA procedures being performed on patients of different ages, it is essential to identify factors that may affect mortality for better patient care.

**Objective:** This study aimed to identify the early mortality rate and potential risk factors among patients undergoing primary total hip replacement (THR) at a tertiary hospital in Tanzania.

**Methodology:** This was a retrospective cohort study conducted from January 2020 to December 2021, which involved patients who had undergone THR.

**Result:** The study involved 183 participants, 53.6% of which were male with a mean age of 55.9 ± 18.4 years. Early mortality (death before 3 months) was found to be 7%. Having hypertension and being seropositive for HIV were independent prognostic factors for survival. Hypertension was associated with an increased chance of death by 4.8 times. The likelihood of death was eleven times higher among participants who were HIV+.

**Conclusion:** Hypertensive patients had an increased chance of death of five times more compared to those with no hypertension. HIV+ patients had an increased chance of death, up to eleven times higher with difference in disease profiles and HIV endemicity in our settings this calls for a different approach to THR.

## 1. Introduction

Total hip replacement (THR) surgery, also known as total hip arthroplasty (THA), is a successful orthopedic procedure becoming more common in developing African countries [1]. However, despite its safety and effectiveness, there are still possible complications, such as pulmonary embolism, dislocation, and infection [2]. Unfortunately, limited information is available regarding THA's epidemiology in developing countries like Tanzania [3].

THA has been found to enhance patient function and improve their overall health-related quality of life compared to traditional treatment methods. Additionally, it is associated with reduced direct costs related to arthritis [4, 5]. However, THA has risks, including the potential for death and various perioperative complications. As medical care has advanced, life expectancy has increased, particularly among patients with long-term systemic illnesses. It has led to a rise in THA procedures among those with comprehensive medical chronic diseases [6, 7].

THA is a well-known procedure that can improve quality of life and functional outcomes and reduce pain despite the potential risks [8]. After undergoing THA, patients may experience complications, with up to 3.9% of cases being affected, and mortality is a concerning outcome [9, 10]. As THA is performed on patients of all ages, it is crucial to identify risk factors associated with increased patient mortality. It is important to note that all medical procedures carry some risk, including the possibility of death. In order to enhance surgical decision making, it is essential to accurately calculate and communicate the risk of mortality following a THA procedure to patients [11].

Furthermore, modifiable surgical risk factors of patients should be identified for preventive steps [11]. The data are scarce in developing countries, including Tanzania. Hence, the study is set to provide this essential information.

## 2. Materials and Methods

This was a single-center retrospective design study on patients who underwent primary THR from January 2020 to December 2021. This study was conducted at the Muhimbili Orthopedic Institute (MOI), a tertiary hospital specializing in orthopedic traumatology and neurosurgery, situated in Dar es Salam, Tanzania.

Ethical clearance to conduct this research was sought from the Institutional Research Ethical review board of the Muhimbili University of Health and Allied Sciences (MUHAS). A permission letter to collect data was obtained from the MOI director's office. Data obtained during the study were kept anonymous.

We included all patients aged 18 years and above who underwent primary THR.

To thoroughly analyze patients who underwent THR from January 2020 to December 2021, the list of patients was obtained from the joint registry book at MOI. Then, the patients' information was isolated from the institution's medical records. The data about mortality were collected from the mortality statistics store of MOI.

Patient files were used to obtain social demographics, clinical conditions (such as diabetes, hypertension (HT), and anemia), and surgical history such as ASA score, mode of anesthesia, and operation time. Any deaths occurring within 90 days of surgery were considered early mortality. For patients who had not returned for follow-up within the last 90 days, their next of kin was contacted to gather information about their well-being.

The collected data were carefully checked and entered into the Statistical Package for Social Science (SPSS) Version 26, released in 2019 by IBM Corp. The checklist was securely kept in a locked cabinet, and a digital copy of the SPSS data file was saved in a computer protected by a password and firewall security.

The checklist data were cross-checked for completeness and consistency to ensure accuracy before entering, cleaning, and analyzing using SPSS. Data cleaning involved checking for missing and duplicate entries. Continuous variables like age and parity were categorized based on their relation to other findings in the literature. Categorical variables were categorized multiple times until final categories were obtained based on class distribution and literature articulation.

Frequencies and percentages were used to summarize categorical variables and we presented them in tables and figures. Measures of central tendency and their respective measure of the spread of data were used to summarize numerical variables.

The success of THR surgery is classified as either surviving or not surviving. Researchers calculate the mortality rate by dividing the number of participants who passed away by the total number of participants and multiplying it by 100. Researchers employ survival analysis to determine the prognostic factors for mortality in THR surgery. This type of analysis classifies the outcome variable as dead or censored (meaning the participant's status is unknown or they withdrew from the study). To examine survival rates, researchers must specify death as the outcome of interest, and the statistical software will group those still alive as censored.

The analysis was conducted using Kaplan–Meier for univariate analysis and Cox regression analysis (hazard ratio) for multivariate analysis. The survival period was measured from admission until 90 days after treatment.

In studying factors that predict mortality after THR surgery, variables with a log-rank *p* value of 0.05 or less were analyzed using Cox regression, a multivariate survival analysis. Hazard ratios were used to determine the influence of these variables on mortality. Variables that were statistically significant at *p* < 0.05 were found to be associated with mortality.

## 3. Results

In this study, a total of 183 participants were involved. The participants' average age was 55.9 years, with a standard deviation of 18.4 years. A detailed breakdown of the social demographic characteristics of the sample can be found in Table 1. It was observed that slightly more males (53.6%) participated in the study than females. Additionally, most participants (60.1%) were below 65 years.

In our study, we found 7% early mortality (death within 3 months after THR). One death occurred in hospital.

Most participants (43.7%) had neck of femur fracture (NOF), while 28.4% had osteoarthritis (OA). 94% of participants were ASA type 1, and regional (spinal) anesthesia was most commonly administered (72.7%), with most surgeries (71.6%) lasting less than 2 hours from cutting time as shown in Table 2. Comorbidities were present in 34 (18.6%) recruited participants, with 11 (32.4%) having multiple comorbidities. HT was the most common comorbidity among 24 (13.1%) study participants.

Among the participants who were diagnosed with HT, 20.8% died within 3 months. On average, patients with HT survived for 84 days.

**Figure 1:**
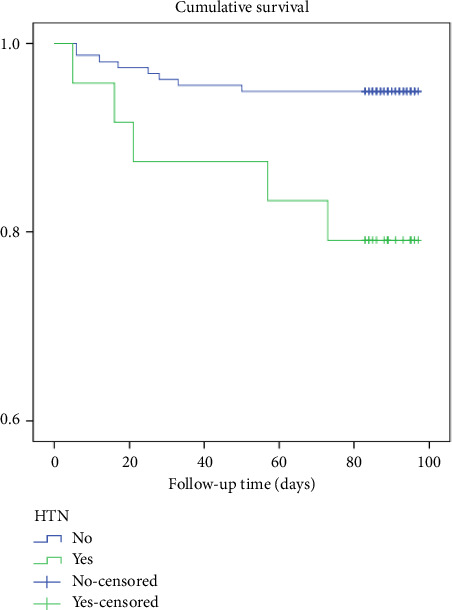
Kaplan–Meier survival plots grouped by hypertensive status and compared by the log-rank test among patients who underwent THR from 2020 to 2021, *N* = 183.

A log-rank test was conducted to compare the survival rates of participants with and without HT. The results showed that the survival rates were significantly different between the two groups, with a *χ*^2^ (1) value of 1.92 and a *p* value of 0.004 (see Figure 1).

**Figure 2:**
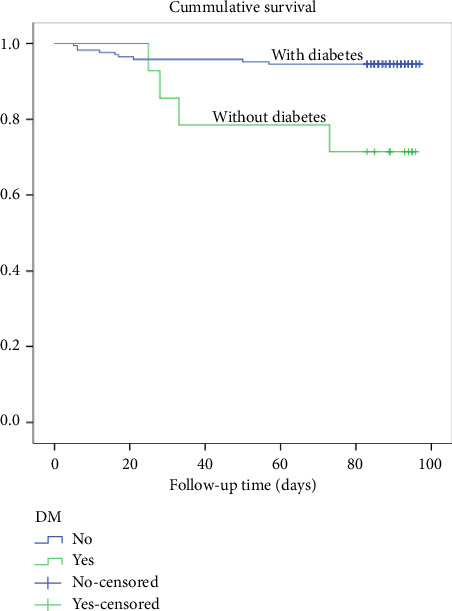
Kaplan–Meier survival plots grouped by diabetic status and compared by the log-rank test among patients who underwent THR from 2020 to 2021, *N* = 183.

A log-rank test was conducted as depicted in Figure 2 to compare the survival rates and it showed a significant difference in survival rates between the two groups, with a *χ*^2^ (1) value of 1.92 and a *p* value of 0.001. Out of all the cases with diabetes, 28.4% resulted in death.

**Figure 3:**
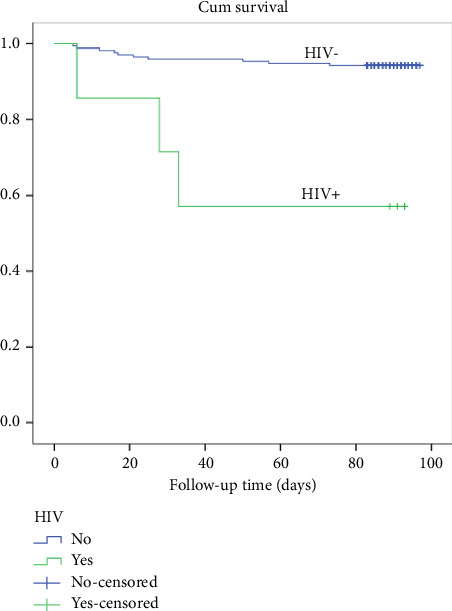
Kaplan–Meier survival plots grouped by HIV status and compared by the log-rank test among patients who underwent THR from 2020 to 2021, *N* = 183.

A log-rank test was run to determine if there were differences in the survival distribution for the different statuses of HIV among participants. The survival distributions for the two having and not having HIV were statistically significantly different, *χ*^2^ (1) = 16.6, *p* < 0.001. Among the total cases of HIV+, 28.4% died (see Figure 3).

**Figure 4:**
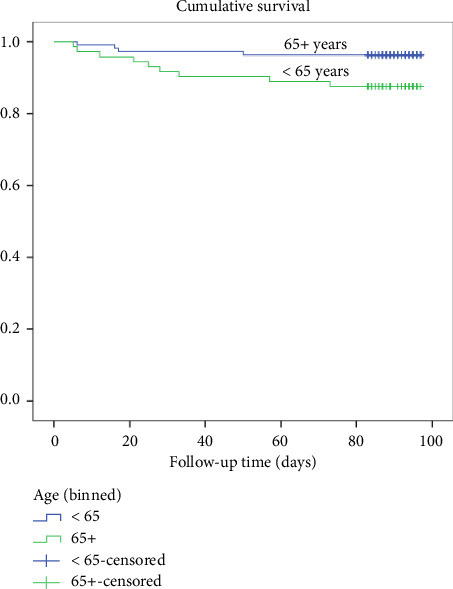
Kaplan–Meier survival plots grouped by age less than 65 years and more than 65 years and compared by the log-rank test among participants, *N* = 183.

A log-rank test was conducted to see if there were any differences in survival rates between those aged less than 65 years and those more than 65 years old. The results in Figure 4 showed a statistically significant difference in survival rates between the two groups, with a *χ*^2^ (1) value of 5.0 and a *p* value of 0.026. The death rate was 3.6% for those under 65 years old and 2.3% for those 65 and above.

**Figure 5:**
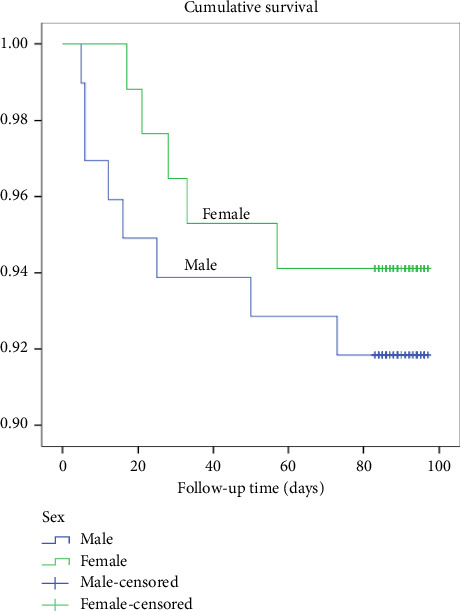
Kaplan–Meier survival plots categorized by gender and compared by the log-rank test among participants, *N* = 183.

There was no significant difference in the survival distribution between gender categories, with a *χ*^2^ (1) = 0.39 and a *p* value of 0.53. The rate of deaths was 8.2% for males and 5.9% for females (Figure 5).

**Figure 6:**
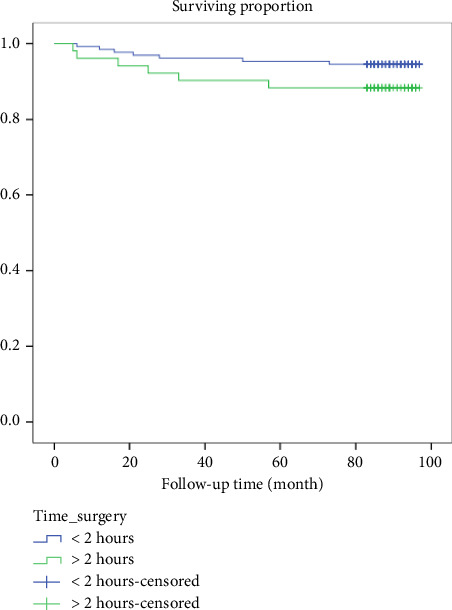
Kaplan–Meier survival plots grouped by categorized time taken to complete procedure and compared by the log-rank test among participants, *N* = 183.

Those who had surgery for more than 2 h had a higher rate of death (11.8%), while those who had surgery for less than 2 h from cutting time had a death rate of 5.3%. A log-rank test was conducted, and the results showed no statistically significant differences in survival rates, with a *χ*^2^ (2) value of 2.42 and a *p* value of 0.3 shown in Figure 6.

**Figure 7:**
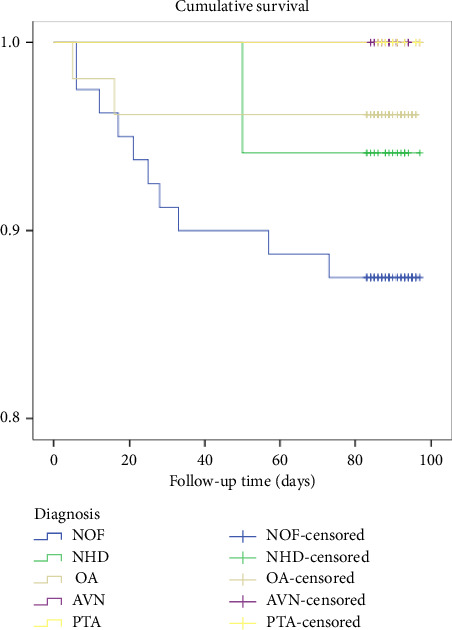
Kaplan–Meier survival plots grouped by diagnosis and compared by the log-rank test among patients who underwent THR from 2020 to 2021, *N* = 183.

Figure 7 shows that death was more among NOF (12.5%), followed by neglected hip dislocation (NHD) (5.9%) and OA (3.8%). A log-rank test was run to determine if there were differences in the survival distribution for the different diagnoses of the participants and the survival distribution among types of diagnosis was not statistically significantly different, *χ*^2^ (4) = 6.84, *p* = 0.14.

**Figure 8:**
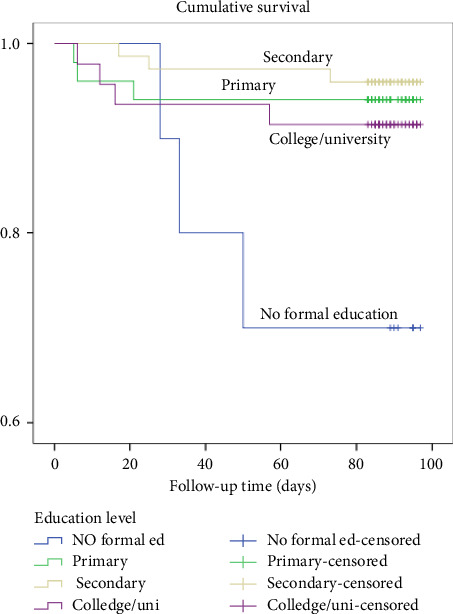
Kaplan–Meier survival plots grouped by education level and compared by the log-rank test among patients who underwent THR from 2020 to 2021, *N* = 183.

The death rate was highest among those with no formal education at 30%, followed by primary education at 5.9%, secondary education at 4%, and college/university education at 7.1%. As shown in Figure 8, a log-rank test showed no statistically significant difference in survival rates among different types of diagnosis, with a *χ*^2^ (3) value of 9.04 and a *p* value of 0.03.

**Figure 9:**
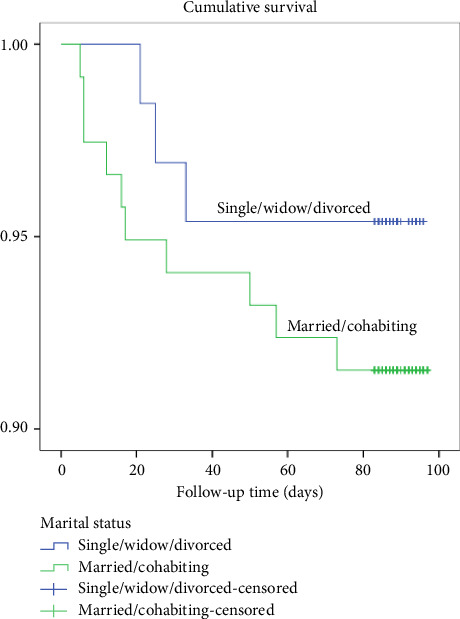
Kaplan–Meier survival plots grouped by marital status and compared by the log-rank test among patients who underwent THR from 2020 to 2021, *N* = 183.

Those who were single, widowed, or divorced had a death rate of 4.4%, while those who were married or cohabiting had a higher rate of 8.5%. A log-rank test showed no statistically significant differences in the survival distribution between the groups, with a *χ*^2^ (1) value of 0.96 and a *p* value of 0.33 as shown in Figure 9.

Table 3 describes the multivariate Cox regression analysis of all variables with significance from the log-rank tests (*p* < 0.05). Having hypertension and being seropositive for HIV were independent predictors of survival. Hypertension was associated with an increased chance of death by up to 4.8 times higher than not having hypertension. The association was statistically significant with adjusted odds ratio (AOR) = 4.791 and *p*=0.015. The likelihood of death was 11 times higher among participants who were HIV+ compared to those who were HIV-negative. There was a statistically significant association with AOR = 10.963 and *p* value = 0.006.

## 4. Discussion

It has been reported that about 25% of postoperative deaths occur after hospital discharge after THR [12]. In this study, 14 (7.7%) died within 90 days after surgery, with one death occurring in hospital before discharge. The two comorbidities that were independently associated with mortality and had the highest attributable fractions were HT and HIV. Hypertensive patients had increased chance of death of five times nonhypertensive. HIV+ patients had increased chance of death of up to eleven times higher than HIV tested negative (Table 3).

The early 90-day mortality rate for patients who underwent THR surgery was higher in this study compared to previous regional studies in countries like Botswana [1] and Burkina Faso [13]. However, it was similar to the rate in a study conducted across 25 African countries [14]. The small sample size of this study may have contributed to the higher mortality rate and could affect the accuracy of the findings. Variations in the study population and methodology may also influence the results. Despite these disparities, the findings are concerning and require further investigation. It is important to note that the study's results may only apply to some populations.

The most common cause of mortality in the immediate postoperative period following THR is cardiovascular disease. In this study, almost 21% of the deaths occurred among hypertensive participants, and hypertensive patients had a hazard ratio five times higher than others. Menendez et al. revealed a 0.2% incidence of myocardial infraction post total joint arthroplasty [15]. Subsequently, all patients with comorbidities were reviewed by physicians preoperatively. Other causes of mortality are pulmonary embolism, cerebrovascular disease, sepsis, and bleeding [8, 16]. The third mortality reported in this study was found to be due to pulmonary embolism. This occurred after the initiation of the physician review program. The cause of death in the Dossche et al. study from Burkina Faso was due to complications of sickle cell disease resulting in massive hemolysis in one patient and pulmonary embolism in another [13]. This highlights the different disease profiles of patients in developing countries compared to developed countries, necessitating different approaches and strategies to the diseases endemic to these countries.

The result of our study found that HIV+ was associated with an increase in mortality among patients undergoing THR surgery by a multiple of eleven (Table 3). A study conducted in Sweden found that HIV+ patients who underwent THR surgery had an 11-fold higher risk of mortality within 90 days of surgery [17]. This highlights the importance of careful patient selection and management in this population to minimize surgical risks. However, a study published in the Journal of Bone and Joint Surgery found that HIV+ patients who received antiretroviral therapy prior to THR surgery had similar complication rates and mortality rates as HIV-negative patients [18]. This suggests that appropriate medical management can mitigate the increased risk associated with HIV+ status in this surgical context.

This study found no significant association between age, gender, and diabetes with mortality rate. However, previous studies have reported a link between advancing age and increased mortality rates. Blom et al. observed a 30-day mortality rate of 0.00% for individuals under 70 years old, 0.048% for those aged 70 to 79, and 1.43% for those aged 80 and above. A study by Belmont et al. identified significant associations between age groups > 80 years or equal and 70–79 years old with increased mortality risk [19]. A systematic review of 32 studies found a consistent observation of male gender as a risk factor for early mortality [20]. Smith, Maru, and Siegmeth investigated the 30-day mortality after elective THA and found a similar association between male gender and higher mortality risk [21]. Throughout the course of this study, no instances of in-hospital mortality were observed.

## 5. Conclusion

On average, one dies in every fourteen patients undergoing THR surgery within 90 days' post-treatment. Hypertensive patients had an increased chance of death of five times nonhypertensive. HIV+ patients had an increased chance of death, up to eleven times higher than patients who tested negative for HIV. With the differences in disease profiles in the developing and developed countries there is a need to approach THR differently in these settings.

## Figures and Tables

**Table 1:** Social demographic characteristics of the patients who underwent THR during the period of 2020 to 2021, *N* = 183.

Variables	Frequencies (percentage)
Sex	
Male	98 (53.6)
Female	85 (46.4)
Age	
< 65	110 (60.1)
65+	73 (39.9)
Marital status	
Single/widow/divorced	65 (35.5)
Married/cohabiting	118 (64.5)
Education level	
No formal	10 (5.5)
Primary	51 (27.9)
Secondary	75 (41.0)
College/university	47 (25.7)
Occupation	
Employed	43 (23.5)
Not employed	85 (46.4)
Self-employed	55 (30.1)

**Table 2:** Treatment and clinical features of the patients who underwent THR during the period of 2020 to 2021, *N* = 183.

Variables	Frequencies (percentage)
Diagnosis	
NOF	80 (43.7)
NHD	17 (9.3)
OA	52 (28.4)
AVN	23 (12.6)
PTA	11 (6.0)
ASA	
Type 1	172 (94.0)
Type 2	10 (5.5)
Type 3	1 (0.5)
Type anesthesia	
Regional	133 (72.7)
General	50 (27.3)
Duration of surgery	
1	131 (71.6)
2	51 (27.9)
3	1 (0.5)
DM	
No	169 (92.3)
Yes	14 (7.7)
HT	
No	159 (86.9)
Yes	24 (13.1)
HIV	
No	176 (96.2)
Yes	7 (3.8)

Abbreviations: ASA, American society of anesthesia; AVN, avascular necrosis; DM, diabetes mellitus; HIV, human immunodeficiency virus; HT, hypertension; NHD, neglected hip dislocation; NOF, neck of femur fracture; OA, osteoarthritis; PTA, post-traumatic arthritis.

**Table 3:** Prognostic factors associated with mortality among patients who underwent THR, *N* = 183.

Variables	95.0% CI for AOR (lower–upper)	*p* value
Age		
< 65 years	Reference	1
65+ years	2.574 (0.615–10.766)	0.195
Education		
Informal	Reference	1
Primary	0.399 (0.064–2.498)	0.326
Secondary	0.307 (0.050–1.890)	0.203
College/university	1.484 (0.213–10.329)	0.690
Diabetes		
No	Reference	1
Yes	1.163 (0.253–5.341)	0.846
Hypertension		
No	Reference	1
Yes	4.791 (1.353–16.968)	0.015
HIV status		
Negative	Reference	1
Positive	10.963 (2.015–59.642)	0.006

## Data Availability

The data that support the findings of this study are available from the corresponding author upon reasonable request.
